# Hypophosphite/Graphitic Carbon Nitride Hybrids: Preparation and Flame-Retardant Application in Thermoplastic Polyurethane

**DOI:** 10.3390/nano7090259

**Published:** 2017-09-05

**Authors:** Yongqian Shi, Libi Fu, Xilei Chen, Jin Guo, Fuqiang Yang, Jingui Wang, Yuying Zheng, Yuan Hu

**Affiliations:** 1 College of Environment and Resources, Fuzhou University, 2 Xueyuan Road, Fuzhou 350116, China; guojin@fzu.edu.cn (J.G.); fqouyang82@163.com (F.Y.); wpjingui@126.com (J.W.); 2 College of Civil Engineering, Fuzhou University, 2 Xueyuan Road, Fuzhou 350116, China; 3 College of Environment and Safety Engineering, Qingdao University of Science and Technology, 53 Zhenzhou Road, Qingdao 266042, China; xilei_chen@foxmail.com; 4 College of Materials Science and Engineering, Fuzhou University, 2 Xueyuan Road, Fuzhou 350116, China; yyzheng@fzu.edu.cn; 5 State Key Laboratory of Fire Science, University of Science and Technology of China, 96 Jinzhai Road, Hefei 230026, China; yuanhu@ustc.edu.cn

**Keywords:** graphitic carbon nitride, inorganic compounds, flame retardancy, smoke suppression, mechanisms

## Abstract

A series of aluminum hypophosphite (AHPi)/graphite-like carbon nitride (g-C_3_N_4_) (designated as CAHPi) hybrids were prepared, followed by incorporation into thermoplastic polyurethane (TPU). The introduction of CAHPi hybrids into TPU led to a marked reduction in the peak of the heat release rate (pHRR), total heat release, weight loss rate, smoke production rate and total smoke production (TSP). For instance, pHRR and TSP decreased by 40% and 50% for TPU/CAHPi20. Furthermore, the increasing fire growth index and decreasing fire performance index were obtained for TPU/CAHPi systems, suggesting reduced fire hazards. It was found that improved fire safety of TPU nanocomposites was contributed by condensed phase and gas phase mechanisms. On one hand, g-C_3_N_4_ accelerated the thermal decomposition of AHPi for the formation of more char layers. On the other hand, g-C_3_N_4_ induced AHPi to generate more free radical capture agents when exposed to flame, besides protecting AHPi against thermal oxidation.

## 1. Introduction

Thermoplastic polyurethane (TPU) has been widely applied due to its outstanding performances, including high tensile strength, abrasion resistance, hydrolytic stability and flexibility [1,2]. TPU consists of soft and hard segments. The soft segment is usually composed of diols, and the hard segment contains diisocyanates and chain extenders. Analogous to other polymers, TPU shows high flammability, and releases a great deal of toxic gas and smoke, coupled with melt-dripping when exposed to flame. These drawbacks limit its further applications. Therefore, it is of great importance and urgency to perform treatment to TPU using highly efficient and environmentally friendly flame retardants.

Halogen-containing flame retardants have proved to be highly efficient in the flame retardancy of TPU at low loadings. Nevertheless, their further applications are prohibited due to the production of many corrosive and toxic gases during combustion [3]. Recently, halogen-free fire retardants have received considerable attention from industrial and scientific communities. Flame-retardant additives (containing phosphorus, nitrogen and silicon) and nanoadditives (clay, layered double hydroxide (LDH), graphite oxide and its derivatives, etc.) have been gradually developed as substitutions for halogenated flame retardants. It is generally accepted that phosphorus-containing flame retardants are endowed with superior flame-retardant efficiency over the others. To date, both ammonium polyphosphate (APP) and aluminum hypophosphite (AHPi) have been regarded as highly effective flame retardants for TPU. A combination of APP and inorganic compounds can lead to remarkable decline in the peak of the heat release rate (pHRR), total heat release (THR) and smoke production. For instance, Chen et al. reported that the addition of 17.5~16.25 wt % APP and 2.5~3.75 wt % ferrite yellow resulted in reduced pHRR and THR by 96 and 78.5%, respectively, along with limiting the oxygen index (LOI) increase from 22.0 to 31.8 vol %, as compared to those of pure TPU [4]. In combination with 2.5 wt % silicon dioxide, 17.5 wt % APP induced LOI to increase to 32.3 vol %, and pHRR and THR to decrease by 91.5% and 69%, respectively [5]. In addition, smoke density was reduced. However, only an UL-94 V-2 rating was achieved. As the content of APP increased to 29.7 wt %, TPU composites could reach UL-94 V-0 rating [6].

AHPi has been extensively utilized in various polymers, because of its high flame-retardant efficiency and environmental friendliness. In contrast to APP, AHPi alone resulted in a slight decline in pHRR (around 30% reduction) at 30 wt % loading [7,8]. It was reported that the LOI value of TPU composites increased to 35.75 vol %, and the pHRR, THR and smoke production decreased by 91.8%, 67.8% and 53.75%, respectively, when 19.94 wt % AHPi was incorporated with 0.06 wt % ionic liquid [Emim]PF_6_ [9]. UL-94 V-0 classification and smoke reduction for TPU composites were achieved by introducing 9 wt % AHPi and 9 wt % melamine cyanurate [10]. Moreover, a combination of AHPi and expandable graphite effectively reduced the pHRR, THR and smoke production of the polymers [11]. Chen et al. investigated the synergistic effect between iron-graphene (IG) and AHPi, and emphasized that LOI of 31.5 vol % and UL-94 V-0, along with the HRR and smoke production rate (SPR) decreasing by 90% and 72%, respectively were obtained by the incorporation of 9.75 wt % AHPi and 0.25 wt % IG [12]. However, AHPi has some drawbacks, such as poor compatibility and a fire risk. When exposed to flame, AHPi produces phosphine as a thermal decomposition product which is combustible and can even evolve into explosive mixtures [13]. Moreover, released heat promotes the further decomposition of AHPi.

Graphitic carbon nitride (g-C_3_N_4_) with a two-dimensional (2D) stacking structure has been widely utilized in catalysts, lithium ion storage, optoelectronic devices, and so forth, because of its excellent thermal, chemical and optical properties [14,15,16,17,18,19,20]. This material contains a small amount of –NH and/or –NH_2_ groups on terminal edges, resulting from incomplete polycondensation [21,22]. These functional groups are considered as anchoring sites which immobilize other components. In comparison with other layered materials, such as graphene, montmorillonite, and LDH, g-C_3_N_4_ is prepared easily, rapidly and cheaply [21,23,24,25,26,27]. In recent years, polymer composites containing g-C_3_N_4_ or its modifications have been studied. The incorporation of g-C_3_N_4_ into sodium alginate led to improved thermal and mechanical performances [28]. A comparative study was performed between polypropylene-grafted maleic anhydride (PP-g-MA) nanocomposites containing g-C_3_N_4_ and functionalized LDH [29]. The results indicated that this novel 2D material could result in superior flame-retardant, thermal, mechanical and ultraviolet light-shielding properties over modified LDH for PP-g-MA nanocomposites. Furthermore, the addition of g-C_3_N_4_/spinel copper cobaltate reduces the fire hazard associated with TPU [30]. Therefore, the combination of g-C_3_N_4_ and AHPi is expected to simultaneously reduce the heat release and smoke generation of TPU.

In this work, a series of AHPi/g-C_3_N_4_ hybrids are prepared through electrostatic interaction, and thereafter added into the TPU matrices to manufacture nanocomposites via a simple melt compounding method. The thermal and fire-retardant properties of TPU nanocomposites are investigated, and the mechanism for TPU fire hazard reduction is also proposed.

## 2. Experimental Section

### 2.1. Raw Materials

Thermoplastic polyurethane (TPU, 85E85) was provided by Baoding Bangtai Chemical Industry Co., Ltd. (Baoding, China). Urea, aluminum sulfate octadecahydrate (Al_2_(SO_4_)_3_·18H_2_O), hypophosphorous acid (H_2_O: 30~35%) and sodium hydroxide (NaOH) were supplied from Sinopharm Chemical Reagent Co., Ltd. (Shanghai, China). The g-C_3_N_4_ was obtained according to the reported work [31]. All chemical agents were used without further treatment.

### 2.2. Synthesis of CAHPi

A series of aluminum hypophosphite/graphite-like carbon nitride (AHPi/g-C_3_N_4_, defined as CAHPi) hybrids were synthesized using a facile mixing approach. Typically, 0.32 g of the obtained g-C_3_N_4_ and 240 mL of deionized water were mixed into a 500 mL of flask fitted with vigorous stirring for 2 h. 6.25 g of hypophosphorous acid was thrown into the suspension above, followed by ultrasonication-assisted agitation for 2 h. The mixture was heated to 85 °C, and pH of the solution was adjusted to 6~7 thereafter. Then 80 mL of 0.16 M aluminum salt solution was added slowly before stirring for 6 h. Finally, the precipitates were washed and dried at 80 °C after filtration. The obtained hybrid was light yellow, which was labelled as CAHPi10. Furthermore, CAHPi5 and CAHPi20, meaning that the weight ratio of g-C_3_N_4_ to AHPi were 5/95 and 20/80, respectively, were prepared by the same approach. For comparison, pure AHPi was synthesized using a similar method without the addition of g-C_3_N_4_.

### 2.3. Preparation of TPU Nanocomposites

The desired concentration of CAHPi*χ* (10 wt % in the work) was incorporated into the TPU matrix to fabricate nanocomposites at 180 °C for 15 min using a Brabender-like apparatus (LH-60, offered by Shanghai Kechuang Plastic Machinery Co., Ltd., Shanghai, China). After blending, these samples were hot-pressed at 190 °C under 5 MPa for 2 min and 20 MPa for 5 min, respectively. The TPU nanocomposites containing CAHPi*χ* were labelled as TPU/CAHPi*χ*, where *χ* was 5, 10 and 20 when CAHPi5, CAHPi10 and CAHPi20 were added, respectively. The same procedure was employed to prepare TPU composite containing 10 wt % AHPi for comparison.

### 2.4. Instruments and Measurements

X-ray diffraction (XRD) patterns were provided by a Japan Rigaku Dmax X-ray diffractometer (RIGAKU, Tokyo, Japan) equipped with graphite monochromatized high-intensity Cu Kα radiation (λ = 1.54178 Å). Fourier transform infrared (FTIR) spectra were performed by a Nicolet 6700 FTIR (Nicolet Instrument Company, Madison, WI, USA). Real-time Fourier transform infrared spectroscopy (RTFTIR) was provided by a Nicolet 6700 FT-IR spectrophotometer (Thermo Scientific, Waltham, MA, USA) equipped with a ventilated oven, which was employed to study the thermo-oxidative degradation of TPU nanocomposites. The RTFTIR spectra of AHPi and CAHPi20 were conducted with a linear heating rate of 20 °C·min^−1^ in the range of 20–600 °C. The morphology of g-C_3_N_4_, AHPi and their hybrids was studied using a scanning electron microscope (SEM) (AMRAY1000B, Beijing R&D Center of the Chinese Academy of Sciences, Beijing, China). Thermogravimetric analysis (TGA) was carried out using a Q5000 thermal analyzer (TA Instruments, New Castle, DE, USA) in the range of 30–800 °C at a heating rate of 20 °C·min^−1^ with a gas flow rate of 100 mL·min^−1^. The flame-retardant additives including g-C_3_N_4_, AHPi and CAHPi*χ* and TPU nanocomposites were performed under N_2_ and air conditions. All these samples were maintained within 5–10 mg. The flammability properties of TPU nanocomposites were assessed via a cone calorimeter (FTT, Derby, UK) according to the ISO 5660/ASTM E1354. Each specimen (100 × 100 × 3 mm^3^) was wrapped in an aluminum foil before radiation by heat flux of 35 kW·m^−2^. The obtained values were averaged. Raman spectroscopy was conducted by a SPEX-1403 laser Raman spectrometer (SPEX Co., Metuchen, NJ, USA) with an excitation wavelength of 514 nm.

## 3. Results and Discussion

### 3.1. Structure and Morphology of CAHPi

**Figure 1:**
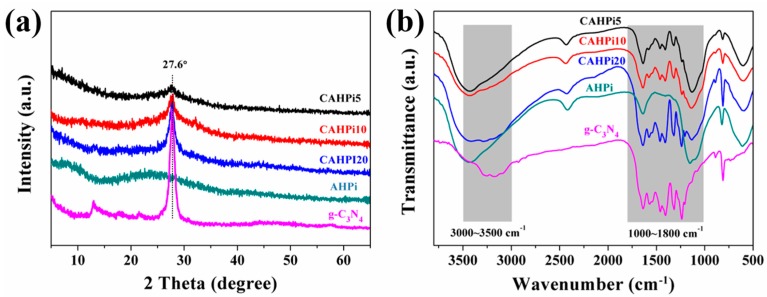
(**a**) X-ray diffraction (XRD) patterns and (**b**) Fourier transform infrared (FTIR) spectra of g-C_3_N_4_, aluminum hypophosphite (AHPi) and their hybrids.

The XRD measurement was employed to study the structural phase of flame retardants. Figure 1a presents the XRD patterns of g-C_3_N_4_, AHPi and their hybrids, showing that a broad peak occurs at 15°–40°, assigned to the amorphous phase of AHPi, while peaks located at 2θ = 27.4° and 13.2° are attributed to the stacking of the conjugated aromatic system and the in-planar repeating unit, respectively, for g-C_3_N_4_ [32]. After the hybriding treatment, a strong diffraction peak corresponding to g-C_3_N_4_ instead of AHPi, is easily observed. Moreover, this peak gradually becomes strong with the increasing weight ratio of g-C_3_N_4_ to AHPi. In order to further verify the coexistence of g-C_3_N_4_ and AHPi, the FTIR technique was adopted to investigate the microstructure of CAHPi, as shown in Figure 1b. For g-C_3_N_4_, the broad bands located at 3000–3500 cm^−1^ are due to stretching vibration of N–H group and hydrogen bonding interactions, and the bands at 1800–1000 cm^−1^ are assigned to stretching vibration of connected units such as C–N(–C)–C or C–NH–C. Furthermore, the absorption band at ca. 812 cm^−1^ corresponds to vibration of the triazine ring [33]. The signals of AHPi are detected at 3400–3500 cm^−1^ (stretching vibrations of the O–H bond in the water of crystallization), 2415 cm^−1^ (stretching vibration of PH_2_), 1153 cm^−1^ (stretching vibration of P=O), 1086 cm^−1^ (symmetric stretching vibration of P–O) and 817 cm^−1^ (rocking mode of PH_2_) [34,35]. It was found that these absorption bands are totally assigned to both APP and g-C_3_N_4_ when g-C_3_N_4_ is combined with AHPi.

**Figure 2:**
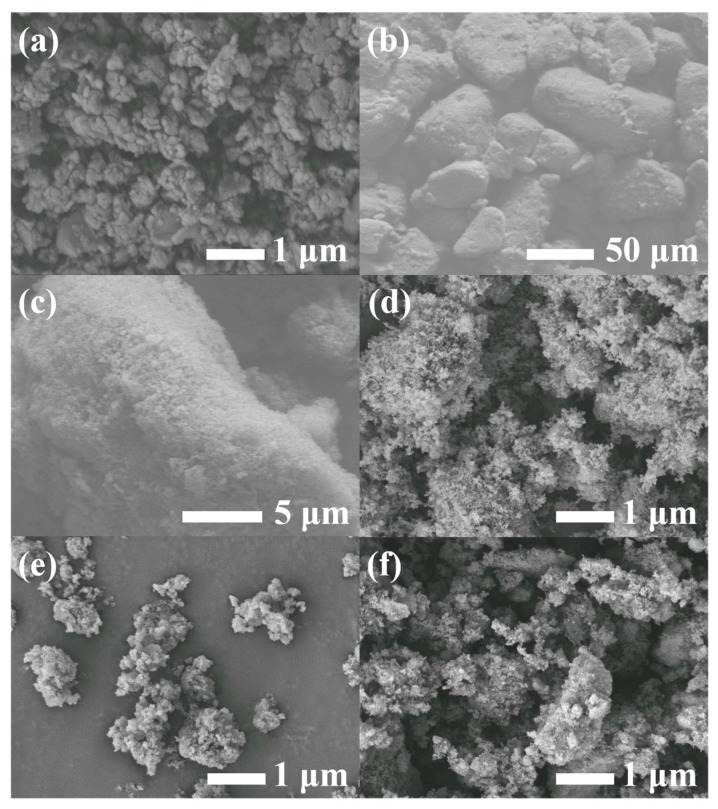
Scanning electron microscope (SEM) images of (**a**) bulk g-C_3_N_4_; (**b**,**c**) AHPi; (**d**) CAHPi5; (**e**) CAHPi10 and (**f**) CAHPi20.

SEM was used to analyze the morphologies of g-C_3_N_4_, AHPi and their hybrids, as depicted in Figure 2. It is clearly observed that bulk g-C_3_N_4_ is composed of solid agglomerates with a layer structure, while AHPi shows an irregular shape with smooth edges (Figure 2a–c). A combination of g-C_3_N_4_ and AHPi leads to their smaller size (Figure 2d–f). It is noted that the surface of these g-C_3_N_4_ nanosheets were coated by a large number of AHPi particles, indicating the existence of synergistic dispersion between the two components.

### 3.2. Thermal Stability of TPU Nanocomposites

**Figure 3:**
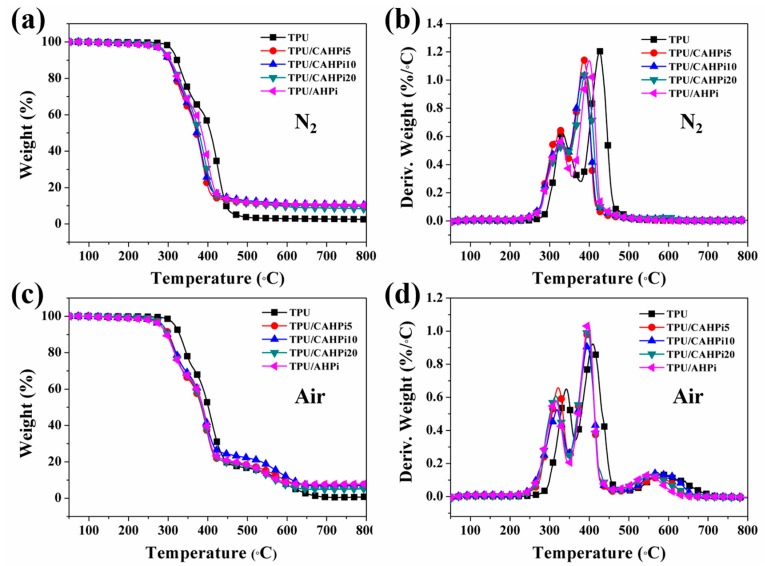
(**a**,**c**) Thermogravimetric analysis (TGA) and (**b**,**d**) derivative thermogravimetry (DTG) curves of thermoplastic polyurethane (TPU) and its nanocomposites in N_2_ and air atmospheres.

The TGA technique has been widely used to estimate the thermal properties of materials. The TGA and derivative thermogravimetry (DTG) curves of TPU and its nanocomposites are plotted in Figure 3, and related data are recorded in Table 1. The initial decomposition temperature and the temperature at the maximal weight loss rate are denoted as T_-10_ and T_max_, respectively. As shown in Figure 3a,b and Table 1, T_-10_ of pure TPU is 322.7 °C under N_2_. It was found that the degradation of TPU is identified as two steps according to T_max1_ = 331.0 °C and T_max2_ = 426.0 °C. The first-step degradation is induced by the breakage of TPU chains, while the second-step degradation is responsible for the further degradation of polyols and isocyanates [36]. In addition, 84.5 and 27.2 wt % of the char residues are obtained at T_max1_ and T_max2_, respectively. When the temperature increases to 800 °C, only 2.5 wt % of the char residues remain. Incorporation of flame-retardant additives into TPU leads to decreased T_-10_ and T_max_, along with increased char yield. However, in the case of TPU nanocomposites, residual content corresponding to T_max1_ decreases, whereas the residual content corresponding to T_max2_ increases, in comparison with those at both T_max1_ and T_max2_ for pure TPU. These results are in good consistence with previous work [8]. Compared with AHPi, the hybrids result in increased T_max1_ and decreased T_-10_, T_max2_ and residual yield. Furthermore, the values of both T_-10_ and T_max_ increase, whereas the content of char residues declines with the increasing weight ratio of g-C_3_N_4_ to AHPi for TPU/CAHPi*χ* nanocomposites.

As illustrated in Figure 3c,d, the thermo-oxidative behavior of TPU and its nanocomposites was measured in air in order to further study the influence of CAHPi on the thermal properties of polymers. It is clearly observed from Figure 3d that TPU exhibits a three-step thermal degradation process. The first two stages, where aliphatic char and volatile products are evolved, are similar with those under N_2_. Compared to char yield corresponding to T_max2_ under N_2_, that under air increases remarkably (43.8 wt %). Nevertheless, the char layer decomposes into an amount of approximately 0.7 wt % residues at 800 °C. The incorporation of the additives into TPU results in a similar trend to those under N_2_ condition. It is worth noting that the combination of g-C_3_N_4_ and AHPi leads to the reduced thermal stability, because g-C_3_N_4_ catalyzes the thermal degradation of AHPi into a great number of phosphorus- and nitrogen-containing chemicals.

### 3.3. Flame Retardancy of TPU Nanocomposites and Mechanism Investigation

**Figure 4:**
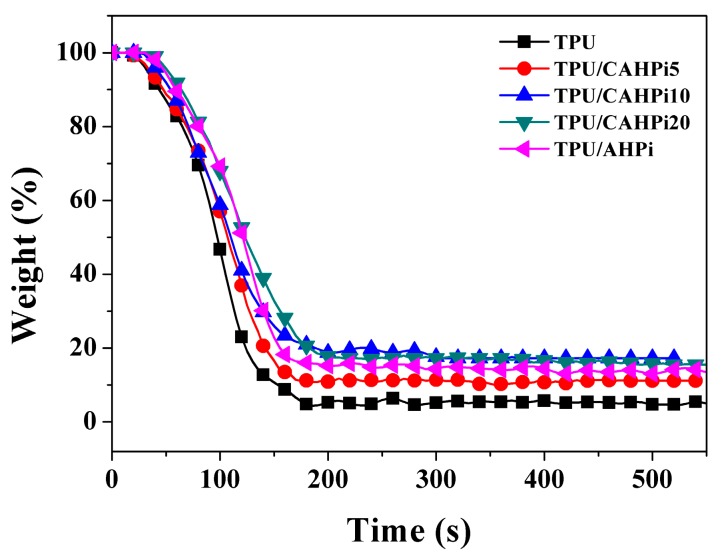
Weight loss of TPU and its nanocomposites during combustion.

The flammability properties of polymeric materials were evaluated by the cone calorimeter, which is often utilized to accurately simulate combustion of materials in a real fire scenario. Figure 4 plots the weight loss curves for all TPU nanocomposites. It is evident that the weight of neat TPU declines rapidly. However, the TPU nanocomposites, especially TPU/CAHPi10 and TPU/CAHPi20, show increased char residues after incorporation of AHPi or its hybrids. An increase in the content of residual char is beneficial for retarding heat and mass transfer. Furthermore, the weight decreases more slowly, indicating a lower weight loss rate.

**Figure 5:**
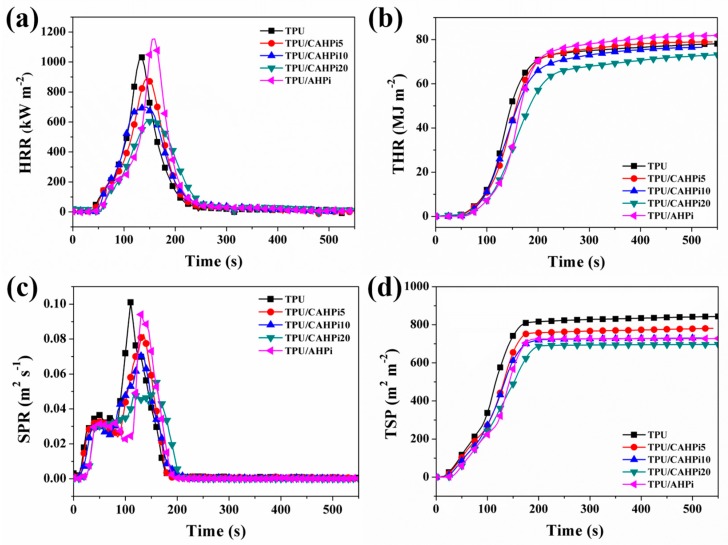
(**a**) Heat release rate (HRR); (**b**) total heat release (THR); (**c**) smoke production rate (SPR) and (**d**) total smoke production (TSP) curves of TPU and its nanocomposites during combustion.

The HRR provided by the cone calorimeter is quite an important parameter as it represents the intensity of a fire. HRR curves of all samples are presented in Figure 5a, and their corresponding data are illustrated in Table 2. Pure TPU burns rapidly after ignition, and shows a sharp HRR curve with peak value of 1031 kW·m^−2^. In contrast, CAHPi hybrids render TPU significantly reduced pHRR. Moreover, the values of pHRR decrease gradually as the weight ratio of g-C_3_N_4_ to AHPi increases. For example, values of the pHRR are reduced by 13%, 32% and 40% for TPU/CAHPi5, TPU/CAHPi10 and TPU/CAHPi20, respectively. Unfortunately, the introduction of 10 wt % AHPi into TPU induces an increase in the pHRR. This result is different from published literatures where the values of the pHRR decreased at loadings higher than 20 wt % [7,8].

The THR is also a key parameter for assessing the fire resistance of a material. It was reported that the gradient of a THR curve could represent flame spread [37]. As observed from Figure 5b and Table 2, the THR values of TPU, TPU/CAHPi5, TPU/CAHPi10, TPU/CAHPi20 and TPU/AHPi are 78.2, 79.1, 78.2, 73.0 and 81.9 kJ·g^−1^, respectively.

Smoke release of flame-retardant materials is an important factor in the field of fire safety. The SPR curves of all the samples are illustrated in Figure 5c. It is apparent that SPR decreases significantly with addition of the fire retardants. The peak value of SPR of neat TPU is 0.10 m^2^·s^−1^ at 110 s during combustion. Incorporation of the flame-retardant additives into TPU leads to reduced SPR. For example, the value of SPR of TPU/AHPi is 0.09 m^2^·s^−1^ at 130 s. In comparison with AHPi, the hybrids result in a further decrease in the value of SPR, especially 0.05 m^2^·s^−1^ at 161 s for TPU/CAHPi20. The total smoke production (TSP) was measured to further assess the smoke release behavior (Figure 5d). The values of TSP of TPU, TPU/CAHPi5, TPU/CAHPi10, TPU/CAHPi20 and TPU/AHPi are 849.7, 781.3, 730.4, 696.9 and 728.2 m^2^·m^−2^, respectively, indicating that TPU/CAHPi20 has the lowest smoke generation among all the nanocomposites.

To identify the fire hazard more clearly, the fire performance index (FPI) and fire growth index (FGI) are adopted. The former denotes the ratio of time to ignition (TTI) to the pHRR, while the latter represents the ratio of the pHRR to the time to the pHRR. Therefore, a smaller value of FPI or larger value of FGI suggests a higher fire hazard of materials [38]. The values of FPI and FGI of TPU and its nanocomposites are listed in Table 2. It is apparent that the fire risk of TPU nanocomposites, especially TPU/CAHPi systems, is much smaller than that of pristine TPU. Furthermore, the flame-retardant system in this work imparts excellent flame-retardant and smoke suppressed properties to TPU at relative low loadings of additives as compared to the previous work (Table 3). These results reveal that AHPi in combination with g-C_3_N_4_ improves the fire safety of TPU. These improvements are ascribed to the explanations that thermal decomposition of CAHPi into enormous free radicals at an early stage accelerates the formation of char, which restrains the penetration of heat and oxygen at a later stage, and the SPR may decrease during the formation of carbon layer.

**Figure 6:**
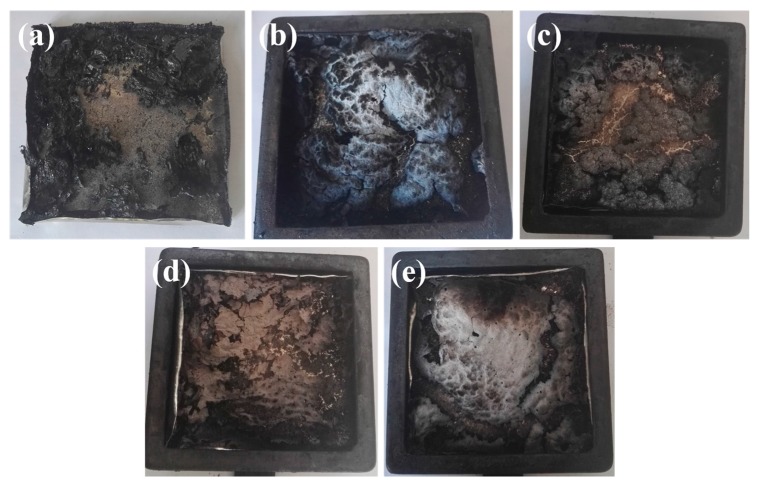
Digital photographs of (**a**) TPU; (**b**) TPU/CAHPi5; (**c**) TPU/CAHPi10; (**d**) TPU/CAHPi20 and; (**e**) TPU/AHPi after the cone calorimeter test.

Figure 6 presents digital photos of char residues for all samples after the cone calorimeter test. An effective carbon layer is able to prevent heat to underlying polymeric materials from a flame zone. It is evident that the char residues of pure TPU are the lightest and loosest, indicative of the highest HRR and largest weight loss among all the samples (Figure 6a). As observed from Figure 6b–e, TPU nanocomposites have a relatively high content of char residues. However, the surface of the char residues from TPU/CAHPi systems is uneven and incompact, in comparison with that of TPU/AHPi composite. It is interesting to observe a conflict where trend of decreasing HRR moderates, whereas quality of char residues becomes good, implying the existence of the gas phase mechanism, besides the condensed phase mechanism.

**Figure 7:**
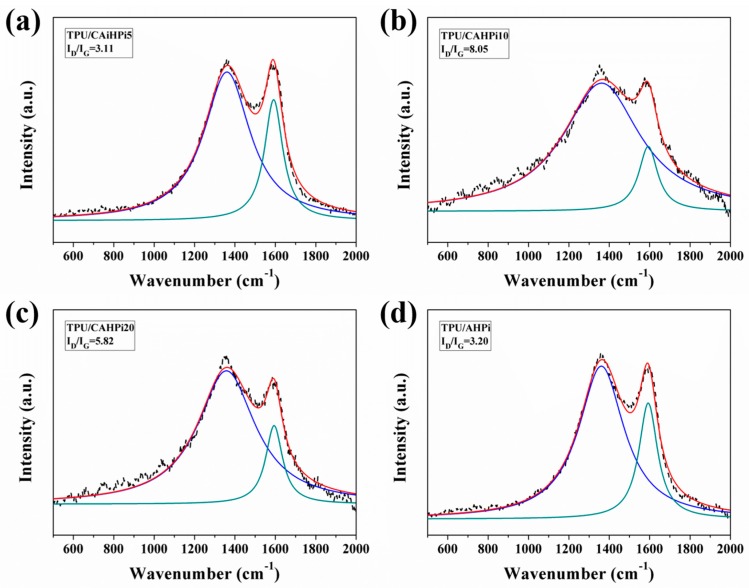
Raman spectra of char residues of (**a**) TPU/CAHPi5; (**b**) TPU/CAHPi10; (**c**) TPU/CAHPi20 and; (**d**) TPU/AHPi. Notes: Black curve represents the real signal; Red curve represents the fitting signal; Blue curve represents the fitting signal at 1350 cm^−1^; Green curve represents the fitting signal at 1590 cm^−1^.

As plotted in Figure 7, the Raman spectrums were employed to study the charring effect of TPU nanocomposites in order to confirm what is deduced above. It was found that all spectra show G and D bands at around 1590 and 1350 cm^−1^, respectively. The graphitization degree of the char can be calculated by an integrated area ratio of D and G bands (*I*_D_/*I*_G_). It is generally accepted that a lower value of *I*_D_/*I*_G_ suggests a higher quality of the char structure. It is evident that the value of *I*_D_/*I*_G_ follows the sequence of TPU/CAHPi5 (3.11) < TPU/AHPi (3.20) < TPU/CAHPi20 (5.82) < TPU/CAHPi10 (8.05), revealing that TPU/CAHPi5 has the highest graphitization degree. This demonstrates that both condensed phase and gas phase mechanisms contribute to marked enhancements in flame retardancy and smoke suppression of the polymer.

**Figure 8:**
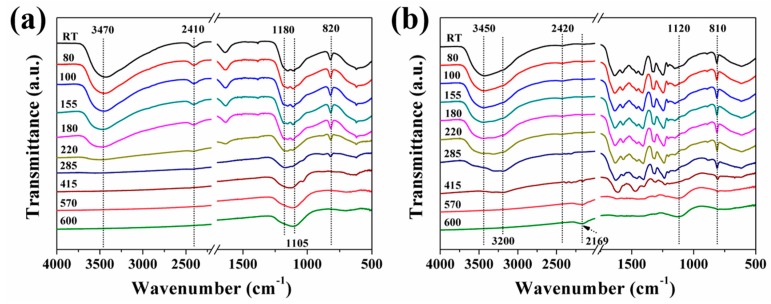
Real-time Fourier transform infrared spectroscopy (RTFTIR) spectra for condensed products of (**a**) AHPi and (**b**) CAHPi20. RT: Real-time.

RTFTIR was conducted to understand evolution of chemical structures during thermally oxidative degradation of flame-retardant additives. The spectra of AHPi and CAHPi20 at different temperatures are depicted in Figure 8. For pure AHPi, two peaks at 3470 and 2410 cm^−1^ corresponding to stretching vibrations of O–H bond in the water of crystallization and stretching vibration of PH_2_, respectively, are visible until the temperature higher than 180 °C. The peak at 820 cm^−1^ (rocking mode of PH_2_) disappears when the temperature rises to 415 °C. The peak at 1180 cm^−1^ assigned to the stretching vibration of P=O, becomes weak, whereas the intensity of band at 1105 cm^−1^, corresponding to the symmetric stretching vibration of P–O, increases gradually upon the temperature exceeding 285 °C. This phenomenon is due to thermal oxidation of metal hypophosphites, contributed by the primary gas-phase degradation of AHPi [39,40]. Compared with pure AHPi, CAHPi20 shows several typical peaks located at 3200, 810 and 1800–1000 cm^−1^, which are assigned to g-C_3_N_4_. It is interesting to observe two absorption bands appearing at 2169 and 1120 cm^−1^ corresponding to –CN evolved from g-C_3_N_4_ and the stretching vibration of P–O, upon the temperature surpassing 415 °C, indicating that oxidative reactions of AHPi are inhibited by g-C_3_N_4_.

## 4. Conclusions

In this work, a series of CAHPi hybrids were prepared, and subsequently used to manufacture TPU nanocomposites through the melt blending method. The microstructural analysis indicated the successful synthesis of CAHPi hybrids. In addition, the synergistic distribution effect between g-C_3_N_4_ and AHPi was observed. TGA results suggested that TPU nanocomposites containing CAHPi had a high content of char residues, resulting from catalytic degradation of AHPi induced by g-C_3_N_4_. The combustion test indicated that the introduction of CAHPi hybrids into TPU led to a marked reduction in the pHRR, THR, SPR, TSP and weight loss rate. For instance, values of pHRR and SPR decreased by 40% and 50%, respectively, for TPU/CAHPi20. Furthermore, increased FGI and decreased FPI for TPU/CAHPi systems demonstrated reduced fire hazards. It was found that improved fire safety of TPU nanocomposites was due to condensed phase and gas phase mechanisms. On one hand, g-C_3_N_4_ catalyzed the thermal degradation of AHPi to generate more char layers. On the other hand, g-C_3_N_4_ prevented AHPi from thermal oxidation, and induced AHPi to release a great number of free-radical capture agents.

## Figures and Tables

**Table 1:** TGA data of TPU and its nanocomposites in N_2_ and air atmospheres.

Sample No.		TPU	TPU/CAHPi5	TPU/CAHPi10	TPU/CAHPi20	TPU/AHPi
N_2_	T_-10_ (°C)	322.7	301.7	302.0	305.2	304.6
	T_max1_ (°C)	331.0	326.5	324.4	333.2	324.4
	T_max2_ (°C)	426.0	386.2	384.1	390.7	399.4
	T_max3_ (°C)	–	–	–	–	–
	Residues at 800 °C (wt %)	2.5	9.7	10.4	8.3	10.2
Air	T_-10_ (°C)	329.0	301.5	298.0	299.2	296.2
	T_max1_ (°C)	342.0	322.2	317.7	315.4	315.4
	T_max2_ (°C)	408.3	397.4	395.2	397.4	397.4
	T_max3_ (°C)	580.7	576.5	571.9	558.7	549.9
	Residues at 800 °C (wt %)	0.7	7.6	7.0	5.3	7.8

**Table 2:** Related cone calorimeter data for TPU and its nanocomposites at 35 kW·m^−2^.

Sample No.	TTI ^1^ (s)	HRR (kW·m^−2^)	THR (MJ·m^−2^)	SPR (m^2^·s^−1^)	TSP (m^2^·m^−2^)	FPI ^2^ (m^2^·s^−1^·kW^−1^)	FGI ^3^ (kW·m^−2^·s^−1^)
TPU	51	1031	78.2	0.10	849.7	0.049	7.637
TPU/CAHPi5	53	896	79.1	0.08	781.3	0.059	6.179
TPU/CAHPi10	59	698	78.2	0.07	730.4	0.084	4.986
TPU/CAHPi20	65	622	73.0	0.05	696.9	0.104	4.013
TPU/AHPi	64	1153	81.9	0.09	728.2	0.056	7.206

^1^ TTI: time to ignition; ^2^ FPI: fire performance index; ^3^ FGI: fire growth index.

**Table 3:** Comparisons in different work on flame-retardant TPU nanocomposites. Peak of the heat release rate (pHRR).

Sample No.	Additives Content (wt %)	pHRR Reduction (%)	TSP Reduction (%)	Ref.
TPU/AHPi	25 wt % AHPi	31%	-	[8]
TPU/AHPi	30 wt % AHPi	27%	−146%	[7]
TPU/AHPi/[Emim]PF_6_	19.94 wt % AHPi + 0.06 wt % [Emim]PF_6_	92%	54%	[9]
This work	10 wt % CAHPi20	40%	50%	-
